# Alteration of peripheral cortisol and autism spectrum disorder: A meta-analysis

**DOI:** 10.3389/fpsyt.2022.928188

**Published:** 2022-07-15

**Authors:** Junwei Gao, Jiao Zou, Ling Yang, Jinghui Zhao, Lian Wang, Tianyao Liu, Xiaotang Fan

**Affiliations:** Department of Military Cognitive Psychology, School of Psychology, Army Medical University, Chongqing, China

**Keywords:** cortisol, autism spectrum disorder, serum, plasma, association

## Abstract

Cortisol is the main HPA axis hormone secreted by the adrenal cortex, and influences metabolism, cognition, and behavior. Recently, a plethora of studies have tried to confirm the correlation between peripheral cortisol and autism spectrum disorder (ASD). However, the results were controversial. We assessed the effects of peripheral cortisol on ASD in this study. The included studies were identified according to the inclusion and exclusion criteria. The pooled Hedges’ g and its 95% confidence interval were selected to evaluate the association between peripheral cortisol and ASD. Subgroup analyses, sensitivity analyses, meta-regression, and publication bias tests were also undertaken based on the obtained information. There were a total of twelve studies with 375 ASD patients and 335 controls included in our meta-analysis. Obvious heterogeneity across studies was found in the overall analysis. Peripheral cortisol levels were significantly elevated in ASD patients compared with controls in the absence of obvious heterogeneity. A single study did not influence the overall comparison results. Meta-regression analyses revealed that age and gender of the included subjects, sample size, and publication year did not moderate effects on the present results. These findings may provide us some targeted strategies to the diagnosis and treatment of ASD.

## Introduction

Autism spectrum disorder (ASD) is a group of neurodevelopmental disorders characterized by impaired social interaction and communication, restricted and repetitive patterns of behavior, interests, or activities according to the Diagnostic and Statistical Manual of Mental Disorders (DSM)-5. The cost of ASD to society is increasing worldwide and is more than $126 billion per year in the United States (1). Data from eleven sites (Arizona, Arkansas, California, Georgia, Maryland, Minnesota, Missouri, New Jersey, Tennessee, Utah, and Wisconsin) in the United States has showed that the overall ASD prevalence was 17.0 per 1,000 (one in 59) children aged 4 years (2). The rising prevalence of ASD is ascribed to complex interactions between genetic and environmental factors (3, 4). However, the exact etiology of ASD remains poorly understood despite of improvements in the diagnosis and treatment of ASD.

The hypothalamic-pituitary-adrenal (HPA) axis is one of the body’s main stress effector systems (5). A compelling body of evidence implicates that the activation of the HPA axis and pulsatile release of glucocorticoids play critical roles in response to physical or psychological stressors in physiological status (6). A growing literature has implicated that the HPA axis contributes to the development of psychiatry disorders, such as depression, anxiety, and PTSD (7–9). Animal studies suggested that the HPA axis dysfunction caused by environmental components could induce autism-like behaviors, and regulation of the HPA axis could alleviate autism-like behaviors (10). Clinical evidence also revealed that the related hormones of the HPA axis were obviously abnormal in ASD patients (11–13). The expression of HPA axis related genes, such as corticotropin-releasing hormone receptor-1 (Crhr1), and oxytocin receptors, were closely related to the development of autism-like behaviors (14, 15). It seems that the HPA axis plays a key role in the occurrence of ASD.

Cortisol is the main HPA axis hormone and the key glucocorticoid produced in response to stress, which has a variety of effects on cardiovascular function, immunity, metabolism, and neurobiology with being involved in several vital biological processes and interactions (16, 17). More and more evidence has shown that cortisol is implicated in emotional and cognitive development, and plays a crucial role in a series of psychiatric disorders (18–20). Cortisol was reported to regulate excitatory and inhibitory (E/I) neurotransmission and neurogenesis during the development of the central nervous system (CNS) (21). Recently, a large number of clinical studies have been performed to evaluate the association between the levels of cortisol in peripheral blood and ASD. However, the results were controversial (22, 23). Therefore, we conducted the present meta-analysis, and pooled the published data to confirm the association between peripheral cortisol and ASD, and strengthen the conclusions.

## Materials and methods

### Publication search

We systematically searched for the relevant literature up through December 21, 2021 in four databases (PubMed, Embase, Web of Science, and Cochrane databases). The retrieval combination of key words was as follows: “autism,” “autistic,” or “ASD”; and “glucocorticoid,” “corticosteroids,” “corticosterone,” “cortisol,” or “cort”; and “serum,” “plasma,” or “peripheral.” We also carried out a reference search on all retrieved records to identify available articles.

### Inclusion and exclusion criteria

Our meta-analysis followed the guidelines recommended by the Preferred Reporting Items for Systematic Reviews and Meta-analysis (the PRISMA statement). Eligible studies must meet all of the following criteria: (1) clinical studies assessing the levels of cortisol in plasma or serum of ASD patients and controls; (2) the levels of cortisol presented as a mean with corresponding standard deviation (SD), or sample size and *P*-value; (3) studies in English. Studies met any of the following criteria were excluded: (1) studies with insufficient data such as concentration of cortisol or *P*-value; (2) animal study or *in vitro* study; (3) case reports, reviews, comments, or meeting abstracts. When some studies contained overlapping data, only the one with the largest sample size was included to calculate the pooled effect size (ES).

### Data extraction

Two independent investigators screened the full text of the retrieved studies according to the inclusion and exclusion criteria, and extracted corresponding data into a standardized Excel spreadsheet. The following information and relevant data were extracted: author, publication year, country, diagnostic criteria, source of ASD patients, detection method for cortisol, sample type, age and gender of samples, sample size, mean, standard deviation, and *P*-value. If a consensus could not be reached, disagreement was resolved by discussion. Finally, data were rechecked by a third author for accuracy.

### Statistical analysis

Hedges’ g is a more unbiased measurement calculated by sample sizes, mean cortisol concentrations, and corresponding SDs, or *P*-values. In our study, the pooled Hedges’ g and its corresponding 95% confidence interval (CI) were employed to evaluate the association between the levels of cortisol in peripheral blood and ASD. Heterogeneity test was conducted to assess heterogeneity across studies using the I^2^-value and Cochrane *Q*-test. A random-effect model was selected to pool the effect size when obvious heterogeneity across studies existed; otherwise, a fixed-effect model was selected. Sensitivity analyses were carried out by removing each study from the analysis in turn to explore the effect of a single study on the result directly. Publication bias was assessed by drawing a funnel plot qualitatively (the more symmetrical, the lower the risk of publication bias), and Egger’s test quantitatively. *P* < 0.05 was considered statistically significant in the present study.

To explore the source of heterogeneity across studies, subgroup analyses were performed based on different types of samples. A Galbraith plot was drawn to identify studies obviously deviated from the baseline. In addition, unrestricted maximum-likelihood random-effect meta-regressions of effect sizes were performed to verify whether some theoretical covariates, such as mean age, gender (male proportion) of ASD patients, sample size, and publication year, would serve as confounders that affect the association of peripheral cortisol and ASD in the present study. All statistical analyses were conducted using Comprehensive Meta-Analysis version 3 software and STATA15.0 software (StataCorp, College Station, TX, United States).

## Results

### Literature search results

**FIGURE 1:**
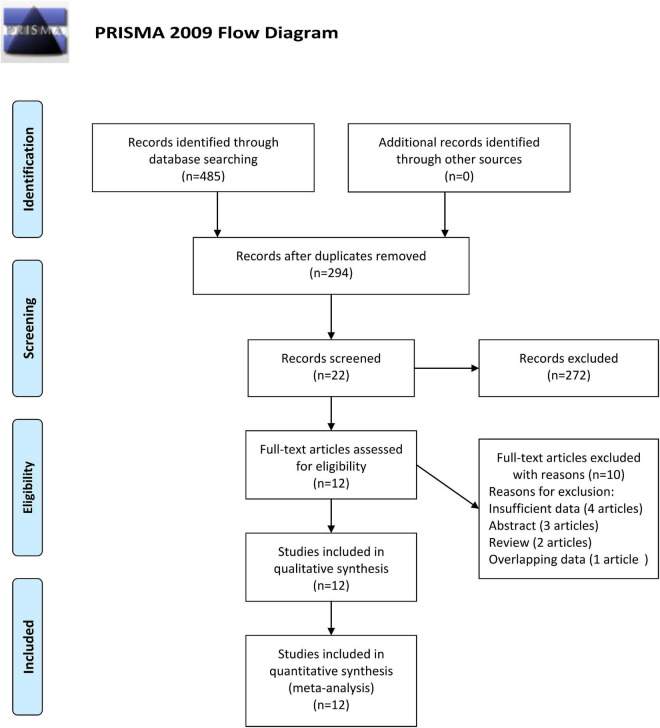
Flow diagram of study identification.

There were 485 studies (126 records from PubMed, 163 records from Embase, 180 records from Web of Science, and 16 records from Cochrane database) identified during the initial electronic search. After removing 191 duplications, there are two hundred and ninety-four studies remaining. There were 272 unrelated records excluded by reviewing titles or abstracts. Twenty-two records underwent further screening through full-text reading. Ten studies were removed for insufficient data, abstract, review, or overlapping data. At last, a total of twelve records (12 studies) met our inclusion criteria and were included in our study (22–33). The flow diagram for the study selection procedure is shown in Figure 1.

### Characters of included studies

Of the twelve included studies, seven detected cortisol concentrations in serum, five in plasma. The studies were conducted in Turkey, Egypt, United States, Japan, Belgium, Finland, Israel, and Croatia. In most of the included studies, the diagnosis of ASD was based on the DSM. We noted that the detection method of peripheral cortisol differed from one another. Most of the included subjects were children, and some were adults. The characteristics of the included studies are presented in Table 1.

**TABLE 1:** Characteristics of the studies included in the meta-analysis.

Sample	First author publication year	Country	Diagnostic criteria	Source	Detection method	Sample size		Cases				Controls			*P*
						Case	Control	Age (years)	Gender (M/F)	Mean ± SD	Unit	Age (years)	Gender (M/F)	Mean ± SD	
Serum	Bozkurt et al. (25)	Turkey	Autism behavior checklist (ABC)	Department of Child and Adolescent Psychiatry in two centers in Turkey	ELISA	33	27	3.98 ± 2.67	33/0	79.1 ± 30.2	ng/mL	3.93 ± 1.01	27/0	60 ± 25.1	0.009
Serum	Hassan et al. (26)	Egypt	The Childhood Autism Rating Scale (CARS)	The outpatient psychiatric clinics of the Neuropsychiatric and Pediatric Departments of the University Hospitals, South Valley University, Qena, Egypt and from the outpatient psychiatric clinics of the Neuropsychiatric Department, Assiut University Hospitals, Assiut, Egypt	ELISA	73	73	7.13 ± 3.52	73/0	5.79 ± 3.63	μg/dL	7.76 ± 4.37	73/0	13.94 ± 2.54	<0.001
Plasma	Corbett et al. (27)	United States	DSM-5	Vanderbilt University	Coated tube radioimmunoassay kits	14	11	9.70 ± 1.93	12/2	0.95 ± 0.11	ng/mL	9.37 ± 1.58	10/1	0.85 ± 0.13	NA
Serum	Iwata et al. (28)	Japan	DSM-IV-TR	The Aichi, Gifu or Shizuoka prefectures of central Japan	ELISA	32	34	12.3 ± 3.2	32/0	74.2 ± 20	ng/mL	12.4 ± 2.6	34/0	58.3 ± 25.3	0.004
Plasma	Hamza et al. (22)	Egypt	DSM-IV	The Institute of Psychiatry and Pediatric Psychiatry Clinic, Children’s hospital, Faculty of Medicine, Ain Shams University, Cairo, Egypt	Chemiluminescent immunometric assay	50	50	7.35 ± 2.6	40/10	11.02 ± 5.23	ug/dL	matched	matched	18.94 ± 3.05	0.032
Serum	Croonenberghs et al. (29)	Belgium	DSM-IV	The outpatient clinic of Child and Adolescent Psychiatry in Antwerp, Belgium; the Mental Health agencies of the same city; and from a Residential Treatment Centre for Autistic Youngsters in Booischot, Belgium	The Bayer Immuno 1 system (Bayer, Brussels, Belgium)	18	22	NA	18/0	85.9 ± 33.5	g/mL	matched	22/0	79.5 ± 34.1	>0.05
Plasma	Tani et al. (30)	Finland	DSM-IV	Department of Psychiatry, University of Helsinki, Finland	Radioimmunoassay kits	20	10	27.2 ± 7.3	14/6	566.5 ± 141.6	nmol/L	26.5 ± 8.1	7/3	619.1 ± 229.7	0.639
Plasma	Strous et al. (31)	Israel	DSM-IV	The Ness Ziona Mental Health Center	The TKCO1 Coat-A-Count kits	15	13	23.6 ± 4.2	11/4	494.08 ± 327.08	nmol/L	30.3 ± 4.1	6/7	447.88 ± 221.15	>0.05
Serum	Ćurin et al. (32)	Croatia	DSM-IV	The Centers for Autism in Split and Zagreb	Standard radioimmunoassay kits	36	27	15.64 ± 9.45	27/9	335.85 ± 131.1	nmol/L	16.62 ± 10.29	19/8	578.56 ± 116.72	<0.001
Plasma	Tordjman et al. (33)	United States	DSM-III-R	Special schools, residential facilities, psychiatric clinics, social service agencies, and parent organizations in the New York City area	Coated tube radioimmunoassay kits	46	23	NA	NA	92.1 ± 41.6	ng/mL	NA	NA	80.6 ± 33.8	NA
Serum	Chew et al. (24)	United States	DSM-5	Stanford University, California, United States	LC–MS/MS	19	18	NA	19/0	83900 (52300–92500)^a^	pg/mL	NA	18/0	89950 (65450–102000)^a^	0.6412
Serum	Spratt et al. (23)	United States	DSM-IV-TR	The Clinical and Translational Research Center (CTRC)	The Bayer ADVIA Centaur Immunoassay System	19	27	NA	NA	13.37 ± 1.10^b^	ug/dL	NA	NA	10.09 ± 0.75^b^	0.014

*^a^Data presented as median (interquartile range).*

*^b^Data presented as Mean ± SE.ELISA, enzyme linked immunosorbent assay; NA: not available.*

### Quantitative data synthesis

**FIGURE 2:**
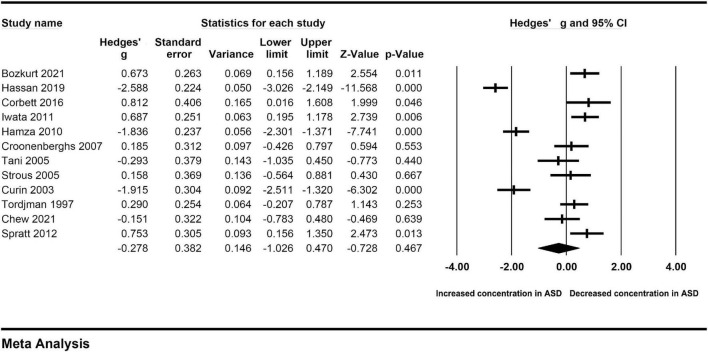
Forest plot for the random-effect meta-analysis.

A random-effect model was selected to calculate the pooled Hedges’ g and its corresponding 95% confidence interval based on the extracted data from the twelve included studies for obvious heterogeneity across studies (I^2^ = 95.198, *P* < 0.001). The combined result of the overall comparison showed that there was no significant difference between the levels of cortisol in peripheral blood of different groups (Hedges’ g = −0.278, 95% CI: −1.026 to 0.470, *P* = 0.467) (Figure 2; Table 2).

**TABLE 2:** Summary of meta-analysis results.

Groups	Studies (n)	case (n)	control (n)	Tests of association				Tests of heterogeneity		
				Model	Hedges’ g [95%CI]	*Z*	*P*-value	*Q*-value	*P*-value	I^2^ (%)
Total	12	375	335	RE	−0.278 [−1.026 to 0.470]	−0.728	0.467	229.091	<0.001	95.198
Subgroups										
Serum	7	230	228	RE	−0.340 [−1.449 to 0.769]	−0.600	0.548	173.869	<0.001	96.549
Plasma	5	145	107	RE	−0.192 [−1.218 to 0.833]	−0.368	0.713	55.204	<0.001	92.754
Removing three studies	9	216	185	FE	0.389 [0.190 to 0.588]	3.833	<0.001	12.102	0.147	33.897

*RE, random-effect model; FE, fixed-effect model.*

### Investigation of heterogeneity

**FIGURE 3:**
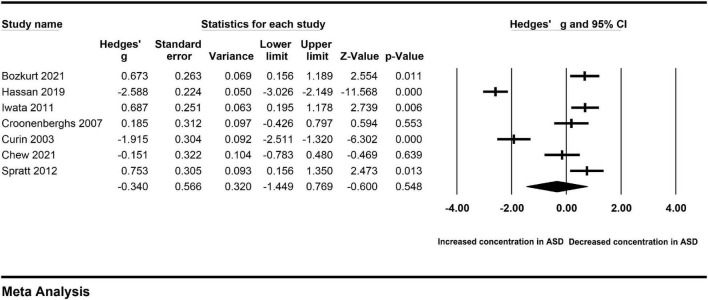
Forest plot for the random-effect meta-analysis of the serum subgroup.

**FIGURE 4:**
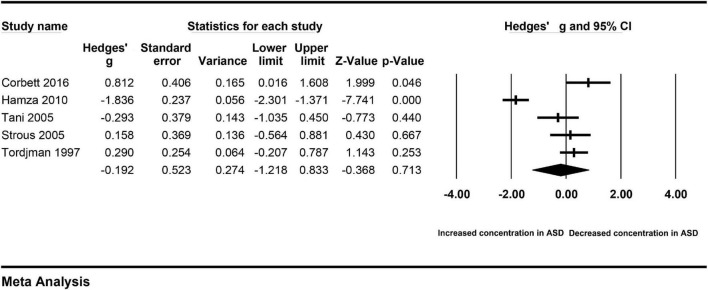
Forest plot for the random-effect meta-analysis of the plasma subgroup.

Obvious heterogeneity may reduce the credibility of the pooled results. To avoid the impact of heterogeneity on the results, the source of heterogeneity across studies was investigated based on the available information. First, subgroup analyses were performed according to the extracted data. There was no significant difference in the levels of cortisol in serum between autistic patients and controls (Hedges’ g = −0.340, 95% CI: −1.449 to 0.769, *P* = 0.548) (Figure 3). Similarly, there was no significant difference in the levels of cortisol in plasma between autistic patients and controls (Hedges’ g = −0.192, 95% CI: −1.218 to 0.833, *P* = 0.713) (Figure 4). However, heterogeneity across studies was not changed obviously either in the serum subgroup (I^2^ = 96.549, *P* < 0.001) or the plasma subgroup (I^2^ = 92.754, *P* < 0.001). The types of samples may not significantly affect the source of heterogeneity.

**FIGURE 5:**
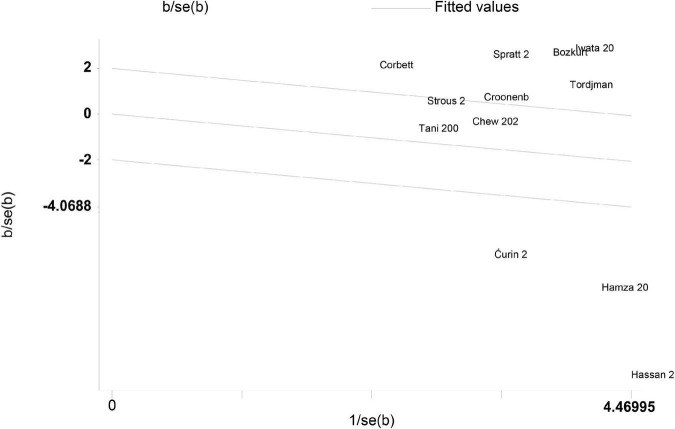
Galbraith plot for the random-effect meta-analysis.

**FIGURE 6:**
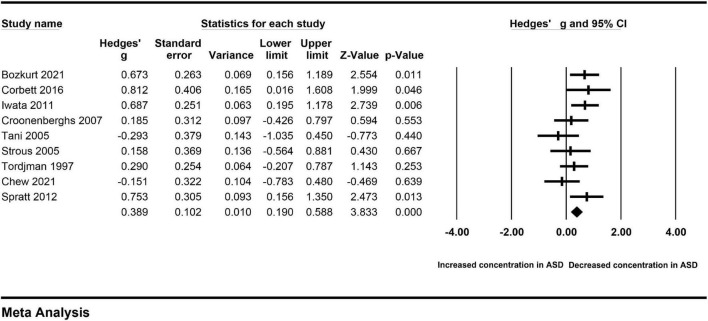
Forest plot for the fixed-effect meta-analysis after removing the studies outside the boundaries of the Galbraith plot.

A Galbraith plot was drawn to identify some specific studies, which may influence heterogeneity across studies. Three studies performed by Ćurin et al. (32), Hassan et al. (26), and Hamza et al. (22) were screened out using the Galbraith plot (Figure 5). These three studies were significantly deflected from the center line, and may be implicated with heterogeneity across studies. Therefore, we removed these three studies, and found that heterogeneity across studies was significantly decreased (I^2^ = 33.897, *P* = 0.147). We also noted that peripheral levels of cortisol were significantly higher in autistic patients compared with controls (Hedges’ g = 0.389, 95% CI: 0.190 to 0.588, *P* < 0.001) (Figure 6). These studies may be a source of heterogeneity across studies, and had a significant impact on the results (Table 2).

**FIGURE 7:**
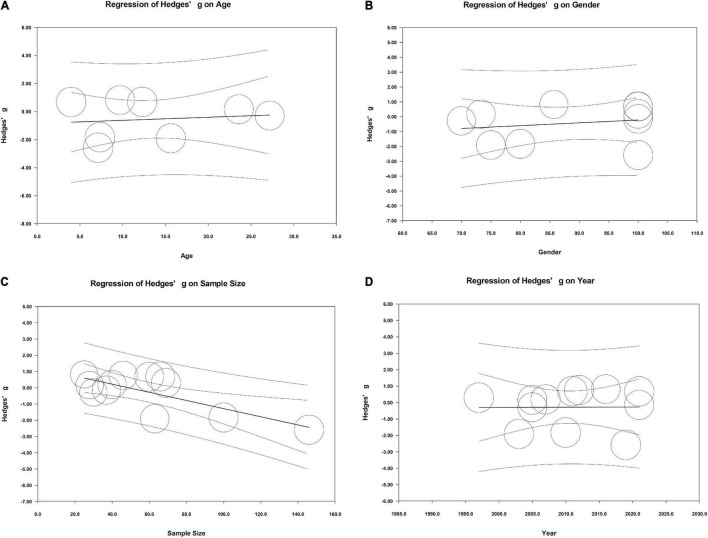
Meta-regression in all studies.

Meta-regression analyses were conducted to evaluate the effects of some potential continuous variables (age, gender, sample size, and publication year) on the high levels of heterogeneity across studies. There were no significant associations between age, gender, sample size, or publication year and the pooled Hedges’ g, which meant that these potential continuous variables did not moderate effects on the outcomes in our meta-analysis (Figure 7).

### Sensitivity analysis

**FIGURE 8:**
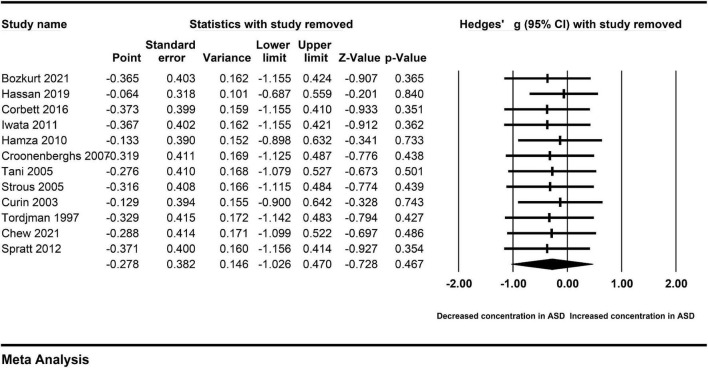
Sensitivity analysis.

To clarify the impact of a single included study on the pooled Hedges’ g, we carried out the sensitivity analysis by removing each included study from the analysis in turn. The association between peripheral cortisol and ASD changed little (Figure 8), suggesting that an individual study did not affect the results.

### Publication bias

**FIGURE 9:**
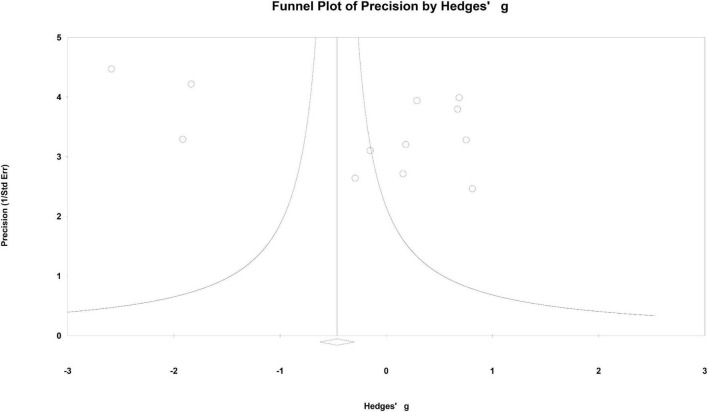
Funnel plot of precision using Hedges’ g statistics.

Visual inspection of a funnel plot suggested that no obvious publication bias was found in this meta-analysis (Figure 9). The results were further strengthened by the result of the Egger test (*P* = 0.156). Moreover, 46 missing studies were required to make the *P*-value less than 0.05, which was confirmed by the classic fail-safe N method. Therefore, publication bias did not affect our results significantly.

## Discussion

This meta-analysis comprehensively evaluates for the first time, to our knowledge, the available evidence regarding the relation of peripheral levels of cortisol to ASD. There were a total of twelve clinical studies included in our meta-analysis to make quantitative evaluation. We found that the levels of cortisol were not significantly increased in peripheral blood of autistic patients compared with controls. The sensitivity analysis was employed to verify whether a single study could affect the overall analysis. When we removed a single study from the overall analysis, the association between peripheral cortisol levels and ASD was not reversed. When there was obvious publication bias in a meta-analysis, the false-negative results were suppressed and the false-positive results could be magnified in this meta-analysis. No obvious publication bias was found in the study, which further confirmed that the results of our meta-analysis were unlikely to be induced by publication bias. More importantly, we noted that obvious heterogeneity across studies existed in the study, which may result in the evidence of relevance association between peripheral cortisol and ASD judged as only low confidence. Therefore, we should be cautious to explain the overall comparative results, and address the issues caused by heterogeneity across studies.

Subgroup analyses, a Galbraith plot, and meta-regression analyses were performed to identify the source of heterogeneity and some potential continuous variables moderating effects on the outcomes. For the limited information, we only carried out subgroup analyses based on different sample types, and found that the sample type was not the main source of heterogeneity across studies. The meta-regression analyses used in the present study confirmed that age, gender, sample size, and publication year may not play a role as moderators in the meta-analysis. Studies with more detailed information are required to explore the source of heterogeneity. The Galbraith plot identified three studies that may cause obvious heterogeneity across studies. After removing these three studies, heterogeneity across studies decreased obviously. Interestingly, peripheral cortisol levels were significantly elevated in ASD patients compared with controls. It means that peripheral cortisol may be associated with ASD. The result of overall analysis may be affected by heterogeneity. The result should be explained with great caution. Future population-based studies are urgently required to verify the effects of peripheral cortisol on ASD.

A clinical study has shown higher hair cortisol concentrations in autistic children with severe self-injurious behavior (34). Fetal cortisol exposure was verified to be a predictor of ASD (35). In addition, more clinical studies have indicated significantly elevated cortisol in peripheral blood of ASD patients (23, 28). What is the relationship between cortisol in peripheral blood and ASD? Early life events are known to greatly influence brain development, and HPA activity. A series of animal studies have suggested environmental factors, such as valproic acid (VPA), maternal immune activation, and maternal separation, resulted in the systemic stress reaction and autism-like behaviors (36–38). As we all know, chronic stress primarily promotes the release of excessive cortisol from the adrenal gland, which tends to activate microglia. Microglia was reported mediating synaptic pruning, which could affect excitation/inhibition imbalance in the CNS. In this way, peripheral cortisol may be partly responsible for the development of ASD (39). Early screening the levels of cortisol in peripheral blood of children may contribute to the diagnosis of ASD. The role of cortisol in the development of ASD, which may help us develop effective interventions on ASD, should be further verified by well-designed animal studies.

In our study, the established methods were used to leverage a large number of records identified from each database searched, and found significantly elevated peripheral cortisol in ASD patients compared with controls after removing three studies causing obvious heterogeneity across studies. Several limitations should be pointed out in our meta-analysis. First, the number of sample size and included studies was moderate, which may influence us to draw accurate conclusions and explore the source of heterogeneity. Second, we should pay more attention to the issues of heterogeneity in our study, although some studies have been found to affect heterogeneity. The inadequate information of included studies blocked our evaluation on some potential residual confounding factors. In addition, subgroup analyses were not fully carried out for the loss of information. Different detection methods investigating cortisol levels in peripheral blood may increase heterogeneity to some extent. Third, the causal relationship between peripheral cortisol and ASD still needs to be treated dialectically. We found that peripheral blood cortisol levels were significantly elevated in ASD patients in the absence of obvious heterogeneity. Whether increased cortisol was a cause of ASD should be further confirmed through more basic research. Finally, the cross-sectional studies could only focus on one point in time of the development of ASD, and the dynamic changes in ASD are still required to be further clarified. Similarly, the dynamic changes cortisol levels should be further monitored.

## Conclusion

We found suggestive evidence that ASD was associated with a significantly increased cortisol in peripheral blood in the absence of obvious heterogeneity across studies. More well-designed studies are required for further confirm the roles of cortisol in ASD.

## Data availability statement

The original contributions presented in this study are included in the article/supplementary material, further inquiries can be directed to the corresponding authors.

## Author contributions

JG and XF conceived the study and corrected the manuscript. JG, JZo, and LY collected the data and drafted the manuscript. JZh, LW, and TL corrected the manuscript. All authors contributed to the article and approved the submitted version.

## Conflict of interest

The authors declare that the research was conducted in the absence of any commercial or financial relationships that could be construed as a potential conflict of interest.

## Publisher’s note

All claims expressed in this article are solely those of the authors and do not necessarily represent those of their affiliated organizations, or those of the publisher, the editors and the reviewers. Any product that may be evaluated in this article, or claim that may be made by its manufacturer, is not guaranteed or endorsed by the publisher.
